# Biosensors for Odor Detection: A Review

**DOI:** 10.3390/bios13121000

**Published:** 2023-11-27

**Authors:** Hongchao Deng, Takamichi Nakamoto

**Affiliations:** Laboratory for Future Interdisciplinary Research of Science and Technology, Institute of Innovative Research, Tokyo Institute of Technology, 4259 Nagatsuta-cho, Midori, Yokohama 226-8503, Kanagawa, Japan; deng.h.aa@m.titech.ac.jp

**Keywords:** biosensor, odorant, olfactory epithelium, olfactory receptor, odorant binding protein, enzyme, aptamer

## Abstract

Animals can easily detect hundreds of thousands of odors in the environment with high sensitivity and selectivity. With the progress of biological olfactory research, scientists have extracted multiple biomaterials and integrated them with different transducers thus generating numerous biosensors. Those biosensors inherit the sensing ability of living organisms and present excellent detection performance. In this paper, we mainly introduce odor biosensors based on substances from animal olfactory systems. Several instances of organ/tissue-based, cell-based, and protein-based biosensors are described and compared. Furthermore, we list some other biological materials such as peptide, nanovesicle, enzyme, and aptamer that are also utilized in odor biosensors. In addition, we illustrate the further developments of odor biosensors.

## 1. Introduction

Odorants are widespread in the atmosphere. The odorants emitted from grain and fruit indicate the spoiled, toxic, or ripe conditions so animals hunt for suitable food [1,2]. Pheromones secreted from one insect can trigger an alarm [3,4], aggregation [5,6], territorial [7,8], or sexual activities [9] in other insects of the same species. A diseased individual releases a distinctive odor so the population can discover or isolate it [10,11]. Besides the natural sources of odorants, industrial activities and man-made objects also produce their unique smells which transmit essential information [12,13,14]. Thus, it is highly demanded that we must detect them precisely in many forms [15,16,17].

The olfactory system of human beings is already sophisticated. However, it is far from enough to detect all important odorants, owing to the insufficient detection range of human olfaction. In order to extend the detection range, detect harmful gases, objectively evaluate the gas type and intensity, and realize automatic measurement, plenty of gas sensors have been developed. Gas sensors can be divided into different categories according to their transducers such as field-effect transistor (FET) [18,19,20], quartz crystal microbalance (QCM) [21,22], surface acoustic wave (SAW) [23,24], surface plasmon resonance (SPR) [25,26], light-addressable potentiometric sensor (LAPS) [27], microelectrode array (MEA) [28,29], and fluorescence [30,31]. The sensing materials, e.g., carbon nanotube, polymer, carbon black composite, conducting polymer, lipid, or ionic liquid are utilized for odorant measurements. These conventional gas sensors have been widely used in our daily lives. However, researchers are still looking for even better sensors.

**Figure 1:**
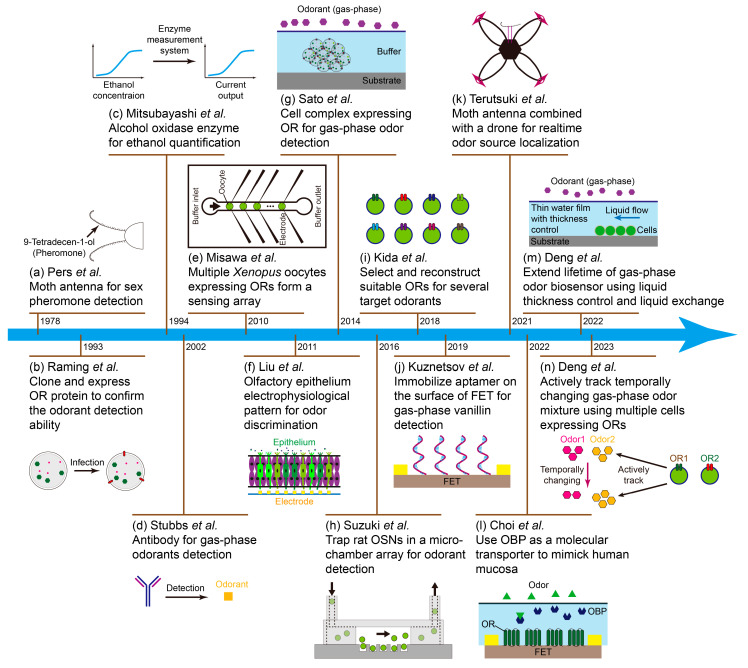
The roadmap of odor biosensors. The corresponding papers are (**a**) Moth antenna for pheromone detection [39]. (**b**) Infect Sf9 cell line with baculovirus harbouring the OR DNA [40]. (**c**) Measure ethanol concentration with enzyme system [41]. (**d**) Antibody for odorant detection [42]. (**e**) Connect multiple oocytes in a fluidic system [43]. (**f**) Record extracellular potentials of olfactory epithelium [44]. (**g**) Cell complex expressing OR for direct gas-phase odorant detection [45]. (**h**) Trap OSNs in a microchamber array [46]. (**i**) Select suitable ORs for target odorants [47]. (**j**) Immobilize aptamer on FET for gas-phase odorant detection [48]. (**k**) Combine moth antenna with drone for odor source localization [49]. (**l**) Use OBP as a molecular transporter for gas-phase odorant detection [50]. (**m**) Extend lifetime of gas-phase odor biosensor using liquid thickness control and liquid exchange [51]. (**n**) Actively track temporally changing gas-phase odor mixture [52].

Besides the sensing materials listed above (we call them conventional materials), scientists fuse the biological materials with the transducers to form odor biosensors. The biological materials are biological substances such as antibodies, enzymes, nucleic acids, cells, epitheliums, nanovesicles, and so on. They also include some synthetic substances similar to biological substances, such as aptamers, peptides, and molecularly imprinted polymers. The biological materials used in odor biosensors are mainly extracted from the creatures’ olfactory systems, for example, olfactory epithelium [32], olfactory sensory neuron (OSN) [33], olfactory receptor (OR) protein [34,35,36], and odorant binding protein (OBP) [37,38]. These odor biosensors have higher sensitivity and selectivity towards their ligands than conventional odor sensors, and also they are not sensitive to temperature and humidity changes. Meanwhile, odor biosensors also encounter several problems such as a short lifetime, low reproducibility, complex operation, high cost, and so on. Thus, many scientists have been working towards better odor biosensors in recent decades (Figure 1).

**Figure 2:**
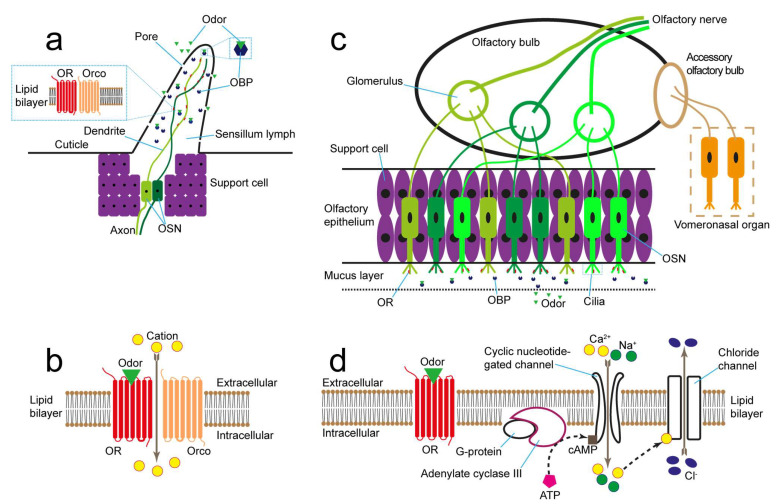
Illustration of insect and vertebrate olfaction. (**a**) Schematic diagram of insect olfactory sensillum. (**b**) Signal transduction procedure of insect olfaction. (**c**) Schematic diagram of vertebrate olfactory system. (**d**) Signal transduction procedure of vertebrate olfaction.

Before introducing the detailed biosensors, we first briefly illustrated the sensing procedures of insect and vertebrate olfaction. Although the biological materials from other creatures such as C. elegans were also exploited in odor biosensors [53,54,55], most biosensor sensing materials were extracted from insects or vertebrates. Insects detect odorants through antennae and maxillary palp. The olfactory sensillum where OSNs exist is distributed along the olfactory organ (Figure 2a) [56,57,58]. The tiny pores on the olfactory sensillum allow odorant molecules to pass through and then dissolve into the sensillum lymph. Odorant molecules are carried by OBPs to OSN dendrites and then are captured by ORs. The ion channel formed by OR and olfactory receptor co-receptor (Orco) opens, resulting in a cation influx, and then a response signal is transmitted along the axon of OSN (Figure 2b) [59,60].

The vertebrate senses volatile odorants through the olfactory epithelium (Figure 2c). When an OR is activated by its ligand, adenylate cyclase III starts to transform the adenosine triphosphate (ATP) into cyclic adenosine monophosphate (cAMP). cAMP opens the cyclic nucleotide-gated channel which leads to calcium and sodium ion influx [61] (Figure 2d). The olfactory signal from OSN is first transferred in the olfactory bulb, and then sent to the brain. The sensing procedures demonstrate that OR and OBP can combine with the odorant molecules. Therefore, the biological materials that contain OR or OBP—for example, olfactory epithelium, OSN, OR protein, and OBP—are suitable for exploitation in odor biosensors.

**Figure 3:**
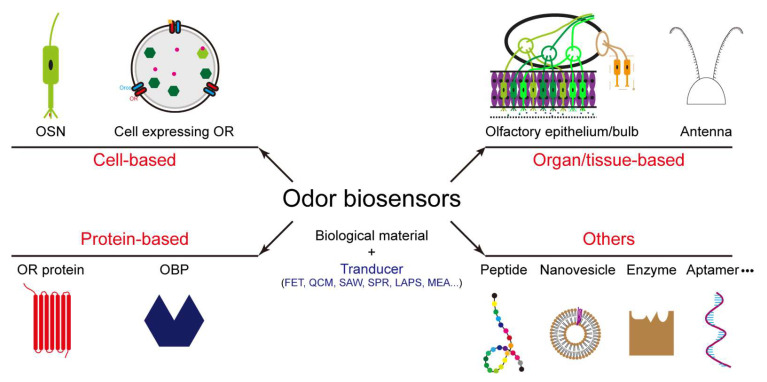
Graphical abstract of introduced odor biosensors in this manuscript.

Many review papers related to odor biosensors have been published before [62,63,64,65,66]. In this paper, biosensors’ principle and their advantages and disadvantages were described, while the previous review papers focused on limited points. Several odor biosensors were introduced based on the sensing materials such as organ/tissue, cell, protein, peptide, nanovesicle, enzyme, and aptamer (Figure 3). We briefly explained their sensing mechanisms and listed corresponding instances. The future developments of odor biosensors were also explained in the final section.

## 2. Organ/Tissue-Based Odor Biosensors

### 2.1. Antenna-Based Odor Biosensor

The antenna is an insect sensing organ for detecting volatile organic compounds (VOCs). In a typical antenna-based odor biosensor, the antenna is usually isolated from silk moth or honeybee [67,68,69]. Two electrodes are connected to the antenna, and the electroantennography (EAG) signal is recorded as the biosensor response.

**Figure 4:**
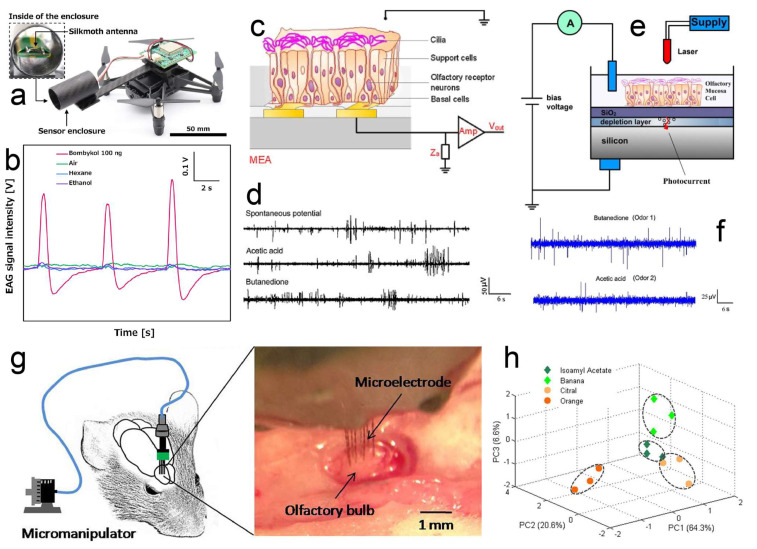
Organ/tissue-based odor biosensors. (**a**) Antenna combined with a drone as a portable biosensor; the voltage signal in the antenna was recorded using a circuit, and then used to control the drone. (**b**) Male silk moth antenna was more sensitive to bombykol than air, hexane, and ethanol. (**c**) Recording extracellular potentials of olfactory receptor neurons in intact epithelium with microelectrodes. (**d**) Tissue electrophysiological signals recorded using microelectrodes after the stimulation of acetic acid and butanedione. (**e**) LAPS system of the olfactory mucosa tissue cells on the sensor surface. (**f**) Tissue electrophysiological signals recorded using LAPS after the stimulation of butanedione and acetic acid. (**g**) Microelectrodes were implanted into the rat olfactory bulb as an in vivo biosensor; the recording region in the olfactory bulb dorsal surface was presented in the figure. (**h**) PCA plot for the classification of isoamyl acetate, banana, orange, and citral. (**a**,**b**) Reprinted with permission from Ref. [49]. Copyright 2021 Elsevier; (**c**,**d**) reprinted with permission from Ref. [72]. Copyright 2010 Elsevier; (**e**,**f**) reprinted with permission from Ref. [71]. Copyright 2010 Elsevier; and (**g**,**h**) reprinted with permission from Ref. [77]. Copyright 2015 Elsevier.

Figure 4a is an example of a male silk moth antenna combined with a drone. The antenna was fixed on a circuit with a 50 Hz sampling rate, and it was more sensitive to bombykol (a kind of sex pheromone) than other odorants such as hexane and ethanol (Figure 4b). Hence, the EAG signal intensity represented the bombykol concentration. The odorant vapor evaporated from the 20 ng/L bombykol sample could induce a visible EAG signal, and the EAG peak emerged within 1 s of odor stimulation. An air pump flew the bombykol odor in a fixed direction to generate a stable odor plume. Based on the EAG signal intensity, the drone adjusted its yaw angle and flew toward the odor source. This bio-hybrid drone is an efficient platform for odor-source localization, and is also appropriate for field VOC detection.

The EAG can also be measured from live insects [70]. In this condition, the sensor lifetime would be much longer, and also it could sense 1 ppb target odorant with a 10 ms response time. However, the immobilization procedure is more complicated compared with only using the isolated antenna, and also the insect movement would introduce additional interfering signals. Therefore, the related research is less recent.

### 2.2. Olfactory-Epithelium- and Olfactory-Bulb-Based Odor Biosensors

The olfactory epithelium (usually rat) can be stripped off from the nasal cavity and cultured in vitro. Its electrophysiological signal relating to cellular functions can be recorded using a MEA or LAPS [44,71,72,73]. When combining the olfactory epithelium with the MEA or LAPS, the basal membrane side contacted the MEA (Figure 4c) or LAPS (Figure 4e) surface, and the cilia side was exposed for odor stimulation. The obtained signals in MEA (Figure 4d) and LAPS (Figure 4f) were potential spikes that looked quite similar. The MEA biosensor can record the multi-channel potential signal simultaneously, thus generating a spatiotemporal pattern of applied odorants [44]. As for the LAPS biosensor, it can detect the potential change on any site of the surface instead of being limited by the position of electrodes. But it only records the potential data in the laser-illuminated place, and also the olfactory epithelium culture on LAPS is more difficult than on the MEA surface.

Besides the in vitro condition, the in vivo sensing can be realized by implanting the microelectrodes into the rat olfactory bulb [74,75,76,77]. Researchers did not choose the olfactory epithelium because the surgery in that area was much more complicated than in the olfactory bulb. For inserting the microelectrodes, the fur over the skull was shaved, the scalp was incised, the skull was removed and then the olfactory bulb, and the related brain area was exposed. According to the received electrophysiological signal in the microelectrodes, the mitral/tufted cell layer was confirmed, and the microelectrodes were chronically fixed onto the rat’s head using dental cement (Figure 4g). After recovering for 4–5 days, this in vivo biosensor can be used for odor detection. The response patterns from all microelectrodes varied among different odorant stimulations. With the proper data-processing method, such as principal component analysis, the classification of four odors could be realized (Figure 4h).

**Figure 5:**
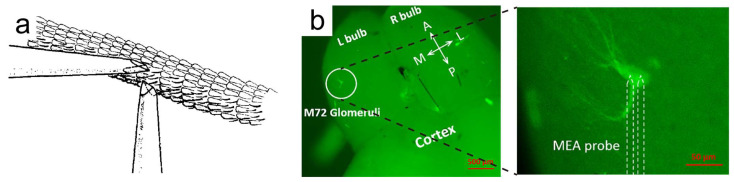
Methods to improve the specificity of organ/tissue-based odor biosensors. (**a**) Schematic drawing of single antennal sensillum measurement. (**b**) M72 OSN-related axons and glomeruli were marked using the green fluorescence. The electrophysiological signal was recorded using an implantable MEA probe. (**a**) Adapted with permission from Ref. [39]. Copyright 1978 Elsevier; (**b**) adapted with permission from Ref. [78]. Copyright 2018 Elsevier.

Since there were multiple types of ORs in an insect antenna or a piece of olfactory epithelium, the electrophysiological signal captured in mitral/tufted cells may come from different glomeruli. To improve the specificity of odor detection, Van Der Pers et al. recorded the single-sensillum EAG signal through an extremely narrow glass capillary (Figure 5a) [39]. Gao et al. used the transgenic technique to handle the experiment’s mice, so the glomeruli that connected with M72 OSN in the olfactory bulb were highlighted with fluorescence (Figure 5b). The electrophysiological signal from this glomeruli could detect lower to a 10^−5^ M liquid sample trinitrotoluene and distinguish trinitrotoluene from other similarly structured chemicals [78]. Another method is using the bioengineering technique to overexpress OR3 on the rat olfactory epithelium and recording the electrophysiological signals from the olfactory bulb [79]. The detection limit could reach around 10^−5^ M towards four ligand odorants.

Among the organ/tissue-based odor biosensors, the sensing materials are easy to obtain. Meanwhile, the responses are to the original electrophysiological signals, and thus could be applied for exploring the creatures’ olfactory systems [80,81]. However, the lifetime of in vitro organ/tissue-based biosensors is relatively short. Although the in vivo organ/tissue-based biosensors extend the lifetime from several hours to several weeks, the surgery is complicated and the experimental results vary among different animals.

## 3. Cell-Based Odor Biosensor

The support cell and basal cell in the olfactory epithelium cannot respond to the target odorants. Also, the recorded electrophysiological signal usually comes from diverse OSNs. Therefore, using dissociated OSN or cell expressing OR as the sensing materials seems more efficient.

### 3.1. Olfactory Sensory-Neuron-Based Odor Biosensors

**Figure 6:**
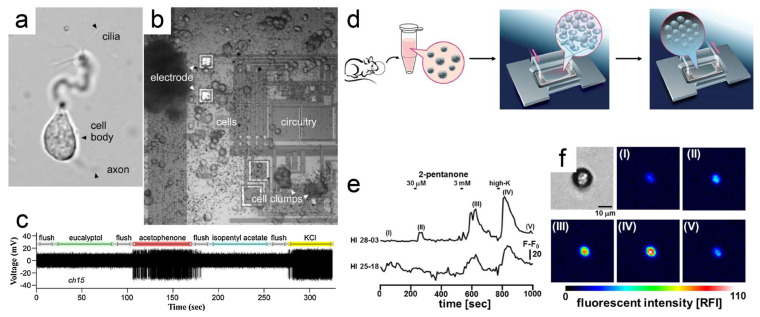
An OSN-based odor biosensor. (**a**) A dissociated OSN from a salamander showing the cell body and several cilia. (**b**) Several OSNs or other cells dissociated from salamander olfactory epithelium plated onto the surface of the chip. (**c**) Response of one electrode channel to the odors. (**d**) The procedure of trapping the OSNs to the microchamber array; the diameter of each well was 10 μm. (**e**) The fluorescent intensity of two Fluo-4 AM labeled OSNs (ID: HI 28-03, HI 25-18). (**f**) The fluorescent images of OSN (ID HI28-03) at each step (I, II, III, IV, and V) of (**e**). (**a**–**c**) Reprinted with permission from Ref. [83]. Copyright 2016 Elsevier; (**d**–**f**) reprinted from Ref. [46].

After dissecting the olfactory epithelium from the nasal cavity, the tissue was treated with papain, trypsin, or other protein enzymes to obtain the isolated cells (Figure 6a). Measuring the potential or labeling with a fluorescent dye (i.e., Fluo-4 AM) and then measuring the fluorescent-intensity change are two methods to detect the biosensor responses [46,82,83]. Since the diameter of OSN was too small to insert the electrodes, recording the potential signal was realized using several planar electrodes (Figure 6b) [83]. Around 10% of the dissociated cells were OSNs, and all the cells were plated onto the chip. The responses from the OSNs meant that close proximity to the sensing electrodes could be recorded, and the selectivity and sensitivity were determined using the ORs expressed on the corresponding OSNs. A circuit was fabricated on the chip to amplify the raw potential signal. The biosensor responses were presented as the increase of voltage spikes (Figure 6c), which was similar to the responses of olfactory-epithelium-based biosensors. Because the OSNs were randomly distributed on the chip, users cannot select the desired OSN type for a specific target odorant.

To solve this problem, Suzuki et al. designed a microchamber array chip [46]. The OSNs were labeled with calcium indicator Fluo-4 AM, added to the flow chamber on the microchamber array, and centrifuged briefly to trap the cells. The Ringer solution was circulated into the chip for removing the floating cells (Figure 6d). When several odorants were applied, the OSNs with responses could be picked out, and their OR types were analyzed. Therefore, this functional high-throughput OSN screening system was more efficient than the planar electrode method. In addition, some scientists cultured ORNs and olfactory bulb neurons together in vitro to sense and discriminate the odor stimulations as well as to serve as a novel model for studying the physiological and pathological mechanisms of olfaction [84].

### 3.2. Cell’s Expressing Olfactory-Receptor-Based Odor Biosensor

In 1989, Jones et al. proposed that the odorant-induced response in the olfactory sensory neuron is mediated by a G protein [85]. To find this protein, they screened the rat olfactory complementary deoxyribonucleic acid (cDNA) library and discovered a protein that related to the olfactory signal transduction [86]. In 1991, Buck et al. cloned and characterized 18 different members of a multigene family that encoded seven transmembrane domain proteins, i.e., OR proteins whose expression was restricted to the olfactory epithelium [87]. Those members were believed to encode a diverse family of odorant receptors. To better investigate the function of OR protein, Dahmen et al. injected the messenger ribonucleic acid (mRNA) isolated from rat or catfish olfactory epithelium into the *Xenopus* Oocytes [88]. After two to six days, the oocyte membrane current sometimes changed with odor stimulations. For enhancing the stability of the OR heterologous expression, Raming et al. transfected the *Spodoptera frugiperda* (Sf9) cell with rat OR DNA (OR5, OR12) via recombinant virus; this was the first time for OR stable heterologous expression [40].

The cells expressing OR has a similar function to OSN. Meanwhile, it can be passaged for many generations with steady characteristics, and the OR types can be freely determined by the researchers [51,52,89,90]. Table 1 lists several examples of cells expressing OR-based odor biosensors. The OR can be expressed on a large cell (such as *Xenopus* oocytes) for easier operation [43] or a small cell (such as HEK293) for higher density [46]. According to the target odorants, researchers can select the sensitive ORs from the OR library [47]. The sensing method can be invasive or non-invasive, simple or completed based on the measurement requirement [43,91].

**Figure 7:**
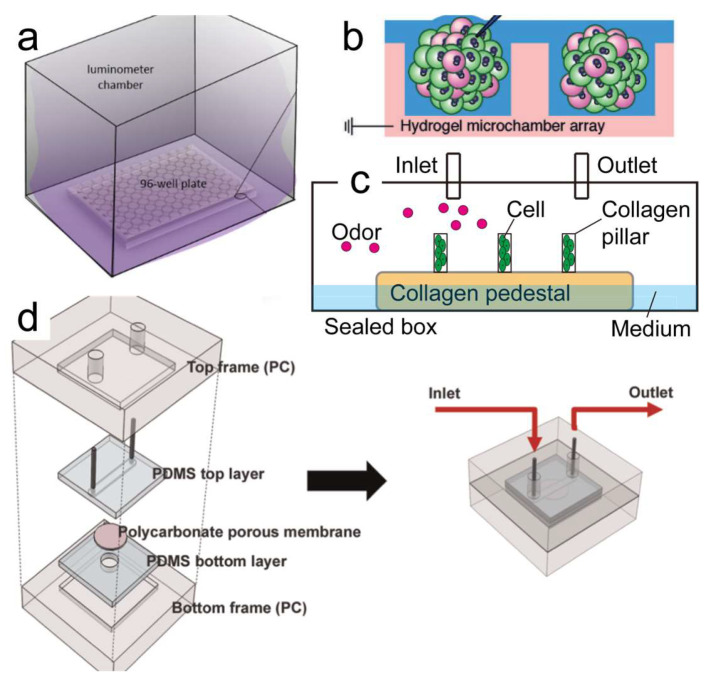
Methods to detect gas-phase odorants. (**a**) The odorant solution was added into the space between the wells of the 96-well plate; the evaporated odorant slowly dissolved into the buffer medium. (**b**) Several spheroids formed by cells were loaded onto the surface of a hydrogel microchamber, and a thin liquid layer covered the spheroids. (**c**) Cells fixed in the collagen pillars, and the buffer medium from the collagen pedestal can prevent the dry-out problem. (**d**) Cells were cultured on the polycarbonate membrane and then assembled into the device; the cell side of the membrane was in contact with the culture medium. PDMS: polydimethylsiloxane. (**a**) Reprinted with permission from Ref. [95]. Copyright 2019 Journal of Visualized Experiments; (**b**) adapted with permission from Ref. [45]. Copyright 2014 John Wiley and Sons; (**d**) reprinted with permission from Ref. [98]. Copyright 2015 Elsevier.

The development of cells expressing OR-based odor biosensors focuses on extending the detection range from the liquid phase into the gas phase and increasing the OR types. Cells require an aqueous environment for maintaining their viability, so these biosensors were developed to detect liquid-phase odorants in the beginning [92,93,94]. Considering that most odorants exist in the gas phase, developing gas-phase odor biosensors is quite important for practical application. There are several methods for gas-phase odorant detection: first was waiting for the gas to naturally dissolve into the buffer medium (Figure 7a), and the buffer volume was enough to do so during the experiment period (>45 min) [47,95]; second was covering the cell with a thin liquid layer (~150 μm), and the time required for odorant molecules to penetrate the liquid film became shorter, thus significantly shortening the response time (Figure 7b) [45,96]; third was fixing the cells into a collagen pillar (Figure 7c), and the buffer medium in the box compensated for the evaporated water from the collagen gel [97]; and fourth was designing a special experiment chamber (Figure 7d), and the cells adhered on a polycarbonate membrane with a 2 μm pore, and the side with the cells touched the culture medium, while the other side was exposed to the gas-phase odorants [98]. Among these methods, the natural dissolution was slow but easy, the collagen pillar and special experiment chamber were complicated but stable, and the response from covering with a thin liquid film was fast but the biosensor lifetime was relatively short (~11 min).

**Table 1:** Examples of cells expressing OR-based odor biosensors.

OR Type	Expressed on	Odorant	Sensing Method	Odorant Concentration	Importance	Ref.
Human OR 5	*E. coli*	Lilial	Fluorescence	0.2–1 mM	Glutathione S-transferase can improve the OR expression level	[99]
Mouse OR-EG	HEK293	Eugenol	Fluorescence	0.01–3 mM	Reconstituted mouse OR in HEK293 cell has a similar detection function to the original one	[100]
Rat I7	Yeast	Octyl aldehyde	Fluorescence	10–50 μM	Screen the proper OR that is sensitive to a specific odorant	[101]
Drosophila melanogaster Or85b	*Xenopus* oocytes	2-Heptanoe	Electrode	10–1000 nM	Build a highly sensitive portable odor biosensor	[43]
Caenorhabditis elegans ODR-10	HEK293	Diacetyl	LAPS	10–100 nM	Label-free functional assays of olfactory receptor	[102]
Rat OR I7	HEK293	Octanal	SPR	0.1–100 mM	Measure molecular interactions in realtime without any labeling	[103]
Caenorhabditis elegans ODR-10	MCF-7	Diacetyl	SAW	10^−10^–10^−4^ mM	Build a highly sensitive odor biosensor	[104]
Rat OR I7	HEK293	Octanal	QCM	10^−8^–10^0^ mM	Find a linear relationship between response and the odorant concentration logarithmic value	[105]
Silk moth BmOR3	Sf21	Bombykal	FET	1–10 μM	Explore the suitable surface for the cell expressing OR	[106]

**Figure 8:**
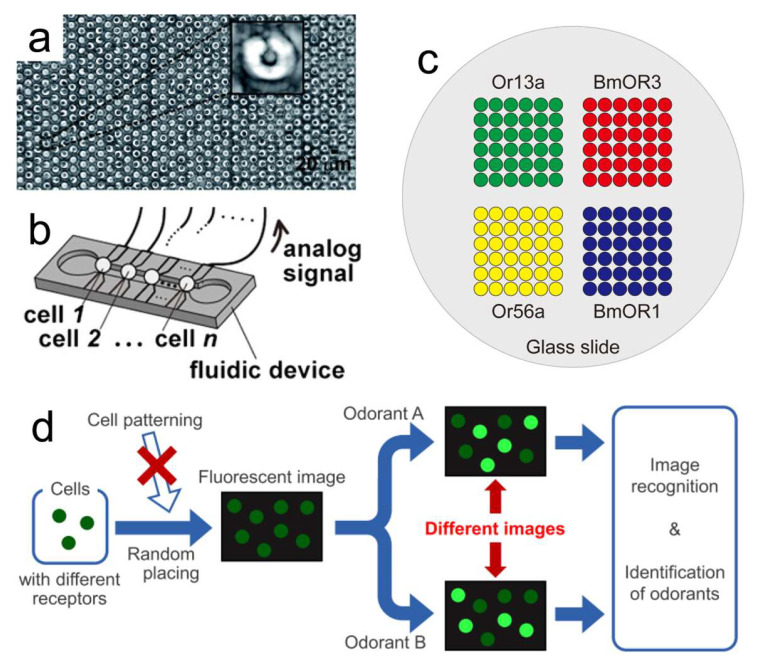
Methods to increase the OR types in odor biosensors. (**a**) A microfluidic microwell array for trapping the cells; the enlarged figure was an OSN in a microwell. (**b**) Multiple *Xenopus* oocyte cells were arranged in a fluidic system. (**c**) The same type of cells was immobilized in the same area. (**d**) Cells were randomly placed, and the cell type was labeled after two single-component odorant stimulations. (**a**) Reprinted with permission from Ref. [82]. Copyright 2010 The Royal Society of Chemistry. (**b**) Reprinted from Ref. [43]. (**d**) Reprinted from Ref. [108].

Increasing the OR types can form a larger sensor array, thereby enhancing the sensing capability. To achieve this target, we can fix the cells in the specific positions. For example, Figueroa et al. produced a microfluidic microwell array to trap different types of OR (Figure 8a) [82]; Misawa et al. arranged multiple cells in a fluidic system (Figure 8b) [43]; and Termtanasombat et al. immobilized the same type of cells in corresponding square areas (Figure 8c) [107]. These operations required pretreatment of the measurement area, but the response data were easy to obtain. We also can mix the cells and then supply several single-component odorants to label the OR types of sensitive cells [108]. In this condition, no pretreatment was required, while the difficulty in data processing increased slightly.

## 4. Protein-Based Odor Biosensor

Although the OSNs or cells expressing ORs can sense the odorants with high selectivity and sensitivity, and most substances in the cells are irrelevant to odorant detection. Therefore, directly using proteins such as OR protein and OBP as the sensing elements is more efficient.

### 4.1. OR Protein-Based Odor Biosensors

The combination of OR protein and its ligands is fundamental to odor detection. There are vast types of ORs existing in various animals such as the pig, honeybee, fruit fly, human, and mosquito [109,110]. For a determined target odorant, we always find one or several ORs that are capable of sensing it. Therefore, OR protein-based biosensors should be the most widely used odor biosensors.

To fabricate an OR protein-based odor biosensor, the first step is obtaining the sensing material. The OR protein concentration in OSN is not so high and obtaining large amounts of OSNs with the desired OR type is difficult. There are two ways to acquire the required OR protein: first is through heterologous protein expression, and second is through cell-free synthesis. In heterologous protein expression, the expression vector containing the OR DNA is established and then introduced into the cells, e.g., *E. coli* and HEK293 for expressing the OR [111,112,113]. The cells are incubated in the culture medium for a period. At this moment, the cells are already suitable for using in cell expressing OR-based odor biosensors. However, for getting the OR protein, these cells are lysed using sonication, and the insoluble fractions are collected, or handled by a membrane-protein-extraction kit to extract the functional protein. As for the cell-free synthesis, researchers only need to add the DNA template and reagents into the device, then it will automatically produce the target protein [114,115,116,117,118]. This method can avoid the issues such as protein aggregation and cytotoxicity which are usually encountered in heterologous expression. However, the generated OR protein is mixed with other components, so centrifugation and purification operations are necessary to reach the final product.

**Figure 9:**
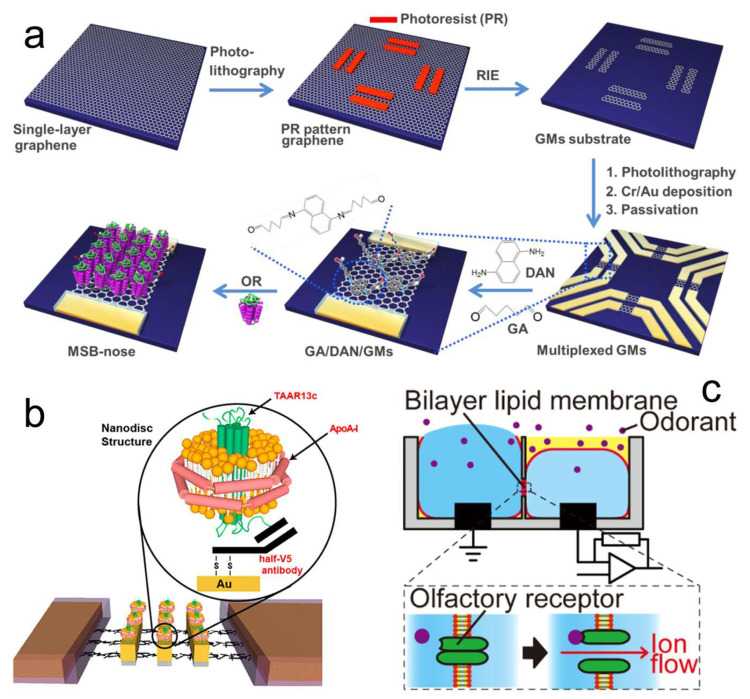
The format of OR protein used in odor biosensors. (**a**) The original format of human OR protein was immobilized on graphene micro patterns (GMs). (**b**) OR protein was inserted into a nanodisc and then immobilized on the carbon nanotube. (**c**) OR and Orco were embedded into a bilayer lipid membrane. (**a**) Reprinted with permission from Ref. [113]. Copyright 2015 ACS Publications. (**b**) Reprinted with permission from Ref. [119]. Copyright 2017 ACS Publications. (**c**) Reprinted with permission from Ref. [124]. Copyright 2019 ACS Publications.

The resulting OR protein can be utilized in the original format (Figure 9a), inserted into a nanodisc (Figure 9b), or embedded into a bilayer lipid membrane (Figure 9c). The original format of OR protein or cell-plasma-membrane fragment was directly immobilized on the transducer surface for capturing the target odorant molecules [111,112,113]. A nanodisc was believed to be more stable than the original OR protein; a nanodisc was composed of a receptor, a lipid bilayer, and membrane scaffold proteins [119,120,121,122]. To construct a nanodisc, the lipids were mixed and solubilized with HEPES buffer, and then the purified OR protein was added, followed by membrane scaffold protein. After incubating for several hours and removing the unbound units, the nanodisc was collected. To embed the OR protein into a bilayer lipid membrane, two kinds of lipid were mixed, and then 1% agarose gel and buffer solution were added, thus forming a bilayer lipid membrane [123,124,125]. Then, the OR protein or OR/Orco complex was added to the bilayer lipid membrane to finish the embedding operation. The basic sensing mechanism of the original OR protein and nanodisc is mass or conformation change. The structure of bilayer lipid membrane with OR is similar to the cell, so the presence of Orco can improve the performance of biosensors [126,127], and also the biosensor response presents as the current change [124].

**Figure 10:**
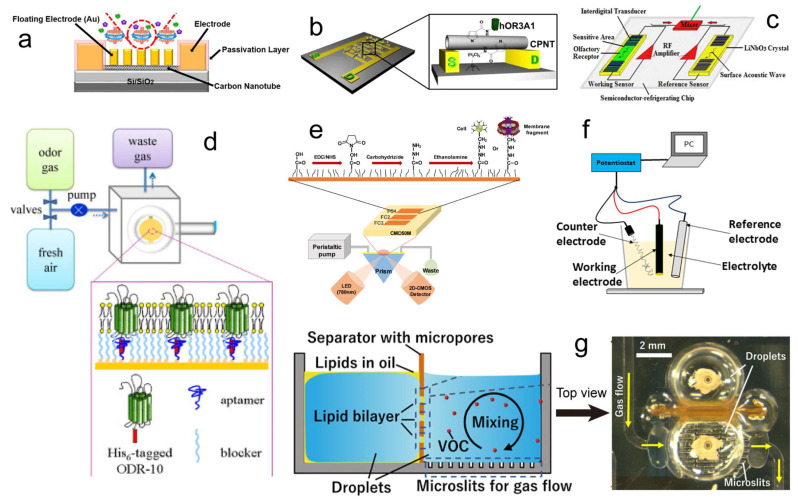
The examples of FET (**a**), interdigitated microelectrode array (**b**), SAW (**c**), QCM (**d**), SPR (**e**), EIS (**f**), and planar electrode pair (**g**) used in OR protein-based odor biosensors. (**a**) Adapted from Ref. [120]. (**b**) Adapted with permission from Ref. [111]. Copyright 2012 Elsevier. (**c**) Reprinted with permission from Ref. [104]. Copyright 2011 Elsevier. (**d**) Adapted with permission from Ref. [112]. Copyright 2013 Elsevier. (**e**) Reprinted from Ref. [128]. (**f**) Reprinted with permission from Ref. [127]. Copyright 2019 Elsevier. (**g**) Adapted from Ref. [125].

The OR proteins can combine with diverse transducers to form odor biosensors. FET [113,119,120,121,122], interdigitated microelectrode array [111], SAW [104], QCM [55,112], SPR [128], electrochemical impedance spectroscopy (EIS) [126,127,129,130], and a planar electrode pair [123,124,125] are all suitable for measuring the biosensor responses (Figure 10). Among them, the research in the last decade is mainly focused on the combination of OR proteins with FET sensors owing to their excellent sensitivity. Unlike the cells expressing OR, which can automatically adhere to the surface of the transducer, researchers should use some immobilization methods for fixing the OR protein. The easier way is using physical absorption, and the protein or membrane fraction is suspended in the solution and then evenly spread on the sensing area of the transducer [55], but the time required is relatively long, and the stability is poor. Another way is using chemical covalent binding, scientists choose the proper material to connect the OR protein and the transducer surface [112]; this method is commonly used most recently because of its high stability [113,119,120,122].

### 4.2. OBP-Based Odor Biosensors

OBP is a small soluble protein that transports the odorant molecules through the aqueous mucus or sensillum lymph [131,132,133,134,135,136,137]. Although the types of OBP [136,138,139,140] are less than OR [141] in the same animal, there are still many OBP-based odor biosensors [142,143,144,145,146,147,148,149,150]. The purification of OBPs is easier than OR proteins because they are secreted into the culture medium rather than remaining in the cell. The transducers and sensing procedures in OBP-based odor biosensors are similar to the OR protein-based odor biosensors, so we will not go into detail here.

**Figure 11:**
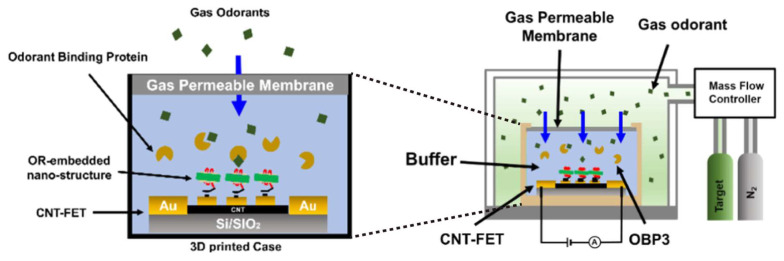
OBP works as a molecular transporter to enhance gas-phase odorant detection ability. Adapted with permission from Ref. [50]. Copyright 2022 ACS Publications.

On the other hand, OBP can be utilized to enhance odorant detection in OR protein-based odor biosensors. Ko et al. inserted the rat OBP3 into a mammalian expression vector pcDNA3 and then transfected it into HEK293 cell [151]. The HEK293-expressing rat OR I7 was sensitive to octanal, and the addition of OBP3 could enhance the responses. A similar conclusion was also proposed by Fukutani et al. regarding using silkworm moth OBP to improve the mouse OR sensitivity [152]. Recently, Choi et al. employed the rat OBP3 as a transporter for insoluble odorant molecules in a buffer medium [50]. The rat OR I7 was embedded into a nanodisc and immobilized on a carbon nanotube FET (CNT-FET). When gas-phase octanal was applied, the existence of OBP can enhance the response of this odor biosensor (Figure 11).

A comparison of different types of odor biosensors is presented in Table 2. We can select appropriate odor biosensors based on the specific measurement targets.

**Table 2:** Comparison of different odor biosensors.

Type	Sensing Material	Advantages	Disadvantages	Ref.
Organ/Tissue	Antenna	Low cost Good sensitivity	Low selectivity Low reproducibility Short lifetime	[39,69]
	Olfactory epithelium/bulb	Low cost Multi-channel data	Complex operation Short lifetime	[72,153]
Cell	OSN	Easy to form a large sensor array Good sensitivity and selectivity	Hard to obtain the desired OSN type Unable to subculture	[46,83]
	Cell expressing OR	Low cost Easy for use Good sensitivity and selectivity Stable characteristic	Hard to obtain a favorable cell line Large individual difference	[47,154]
Protein	OR protein	High sensitivity and selectivity Easy to combine with transducers	Hard to purify High cost	[119,125]
	OBP	Easy to purify Good sensitivity Easy to combine with transducers	High cost Low selectivity	[147,150]

## 5. Other Biological Materials for Odor Biosensors

Besides the aforementioned sensing materials, some other biological substances that can specifically bind to odorant molecules are also suitable for odor biosensors.

**Figure 12:**
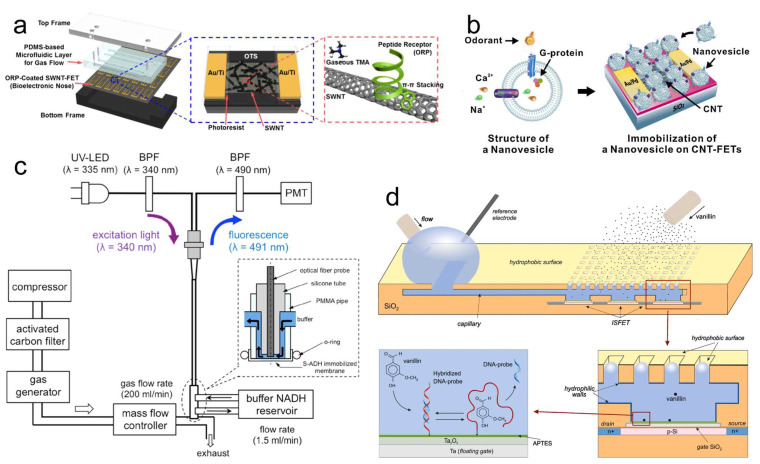
Other biological materials for odor biosensors. (**a**) Peptide-based odor biosensor; peptides derived from OBP were assembled with a PDMS-based microfluidic layer between top and bottom frames. (**b**) Nanovesicle-based odor biosensor; the nanovesicle was generated from the cell expressing OR, and it had the same membrane proteins and cytosolic components as the original cell, while the size was much smaller. (**c**) Enzyme-based odor biosensor; the enzyme was immobilized on the flow cell, and the fluorescent-intensity change indicated the concentrations of gaseous acetone. (**d**) Aptamer-based odor biosensor; when vanillin combined with the aptamer on the ISFET, the surface potential increased. (**a**) Reprinted with permission from Ref. [158]. Copyright 2015 Elsevier. (**b**) Reprinted with permission from Ref. [160]. Copyright 2012 Elsevier. (**c**) Reprinted with permission from Ref. [162]. Copyright 2015 Elsevier. (**d**) Reprinted with permission from Ref. [48]. Copyright 2019 Elsevier.

First are the peptide-based odor biosensors. Although the OR protein or OBP are proper sensing materials for high sensitivity and selectivity odor biosensors, the manufacturing and purification processes of those proteins are complicated and labor intensive, also maintaining the stable structures of these proteins is relatively difficult. Considering that the part that binds the target odorant molecule is only a tiny portion of protein, using the OR- or OBP-derived peptide is a more effective solution for odor biosensors [155,156,157]. Lim et al. manufactured a single-walled-carbon nanotube FET (SWNT-FET) functionalized with OR-derived peptides (Figure 12a). Functionalization was performed using the property of SWNT for which aromatic rings were stacked on the surface using π–π interactions [158]. This biosensor was able to sensitively and selectively detect trimethylamine at a concentration of 10 fM and discriminate TMA from other similar molecules in real-time. In addition, peptides can be freely designed by the researchers. Homma et al. [159] designed two peptides and immobilized them, as well as a molecular scaffold peptide, on the graphene surface to form three FET biosensors. These biosensors could detect 10 pM limonene and discriminate different odorants. But, the desorption was not so successful after odor stimulation.

Second is the nanovesicle-based odor biosensors. Nanovesicle is secreted from the cell for intercellular communication. The protein, DNA, and RNA in the nanovesicle are the same as the original cell. Therefore, a nanovesicle from a cell heterologously expressing OR also has the same OR protein on its membrane and could be exploited for odor detection [160,161]. Jin et al. transfected the HEK293 cell with human OR 2AG1 and then collected the nanovesicle (Figure 12b) [160]. Because the OR protein was embedded in the bilayer lipid membrane and the nanovesicle was much smaller than the original cell, the OR function was stable and the nanovesicle could be easily combined with CNT-FET. This biosensor could sense amyl butyrate at 1 fM concentration. With the handling of fluorescent dye Fura 2-AM, the biosensor-response signal is also presented as fluorescent-intensity change.

Third is the enzyme-based odor biosensors. In reference [162], a flow-cell with nicotinamide adenine dinucleotide (NADH)-dependent secondary alcohol dehydrogenase (S-ADH) immobilized membrane was attached onto a fiber-optic NADH measurement device to form the fiber-optic biochemical-gas-sensing system (Figure 12c). The enzymatic reaction of acetone and NADH was evaluated using fluorescent-intensity change, and this system could measure the acetone gas from 20 to 5300 ppb.

Fourth is the aptamer-based biosensors. Aptamer can be RNA, single-stranded DNA, or double-stranded DNA. The combination of the aptamer and target molecule is similar to that of antigen–antibody. In reference [48], the aptamer was immobilized onto the surface of an ion-selective FET (ISFET). When vanillin odorant was applied, it diffused through the pores and dissolved into the buffer medium, and then was captured by the Van74 DNA aptamer (Figure 10d). Vanillin molecules replaced the hybridized probes on the sensitive surface resulting in an increase of surface potential, and this biosensor can detect vanillin over a concentration range from 2.7 ppt to 0.3 ppm.

Some other materials such as the taste-receptor protein [163,164] and antibody [42] were also employed for odor biosensors. Due to the space limitation, we cannot enumerate all types of odor biosensors here. The selection of biological materials should be determined based on the actual application situations.

## 6. Conclusions

The trends of odor biosensors are more efficient and compact, have higher sensitivity and selectivity, and a wider detection range. Tissues/organs, cells, and proteins are all capable of being the sensing materials of odor biosensors, but the smaller size is easier for combining with transducers, and the non-sensitive substance is less. The peptide seems to be the ultimate form of sensing material owing to its simplicity, diversity, and ease of production. The design of peptides could be inspired by the working area of the OR protein or OBP, and is also freely determined by scientists. Therefore, the latent detection ability of peptide should be higher than that of the existing OR protein or OBP. The sensitivity and selectivity of biosensors are mainly determined using the sensing material, while they are also affected by the transducer. Hence, the OR and OBP libraries are established for researchers to select the optimal sensing material for the specific ligand [165]. Meanwhile, the high-sensitive transducers, e.g., CNT-FET and QCM have been commonly used recently. The nanomaterials, such as the nanowire [166,167], nanoparticle [168,169,170], and nanotube [171,172], that have a large surface area to volume ratio can improve the sensitivity of biosensors, thereby becoming well-developed in recent years. Most odor biosensors work in the liquid phase, while most VOCs exist in the atmosphere. To extend the detection range, accelerating the VOC dissolution [173,174,175] and direct gas-phase odorant detection, there are two optional methods.

## 7. Future Perspectives

Although many odor biosensors have been developed, odor sensing towards complex odor mixtures has rarely been discussed. With the data from OR response towards odor mixture, we can build a response model that enables us to predict the OR responses under other odor stimulations, and finally predict the response of the animal olfaction to natural odorants, especially complex mixtures of numerous molecules.

The types of OR in one odor biosensor were usually less than four [176,177], which is much less compared to the OR types in animals. Meanwhile, a biosensor usually contains only one transducer. These facts result in the detection ability of current odor biosensors being completely unable to match animal olfactory perception [178]. A sensor array which consists of a set of sensors with various sensing materials can overcome the individual differences in biological material, has a more powerful sensing capability, and could be utilized for reconstructing the animal olfactory system [179]. Thus, manufacturing a large sensor array and through fusion of diverse transducers we are expected to improve biosensor performance [180]. In Figure 8, different types of cells expressing OR are put together to enhance the biosensor detection capability. Other biological materials such as aptamer, enzyme, OR protein, or OBP could also be assembled together to generate a sensor array. Also, diverse transducers could be connected in parallel (the unknown odor to be measured is separated into different channels and each channel has one transducer) or in serial (there is only one channel and all transducers are installed in this channel). In this condition, the biosensor is capable of mimicking the natural olfactory system [179]. On the other hand, the dimensions of the gathered experiment data will be much larger than the current situation [180]. Hence, we need machine learning methods for pattern recognition to gain insight into complex data. The machine learning methodologies are mainly utilized in three types of tasks: classification, clustering, and regression [181,182,183]. Scientists can effectively detect the key parameters or hidden patterns with the assistance of machine learning. The study of odor biosensors is still in the early stages. With the deepening of research, more powerful biosensors will be developed to contribute to our daily lives.
